# Supplementary material for: A Multi-Layer Breast Cancer Model to Study the Synergistic Effect of Photochemotherapy
Source: Micromachines (Basel). 2023 Sep 21;14(9):1806. doi: 10.3390/mi14091806 (PMC10535669; doi:10.3390/mi14091806)
Supplement: Supplementary file 1 [file micromachines-14-01806-s001.zip › micromachines-2601132-supplementary.pdf]

A Multi-Layer Breast Cancer Model to Study the Synergistic Effect of Photochemotherapy

Magdalena Flont [1,2] and Elżbieta Jastrzębska [1,2,*]

[1] Faculty of Chemistry, Warsaw University of Technology, Noakowskiego 3, 00-664 Warsaw, Poland; magdalena.flont@pw.edu.pl
[2] Center for Advanced Materials and Technologies CEZAMAT, Warsaw University of Technology, Poleczki 19, 02-822 Warsaw, Poland
[*] Correspondence: elzbieta.jastrzebska@pw.edu.pl; Tel.: +48-22-234-7253

Supporting information

1. The cell introduction into the microsystem and 3D cell culture creation

Comparison of different cell seeding density ratios in co-culture

In order to obtain a three-dimensional and multilayer cell culture in the developed microsystem, it was necessary to optimize the ratio of the density of non-malignant cells and cancer cells introduced into the microsystem. To prepare the cell suspension for tests in the microsystem, the fibroblasts (HMF) and cancer cells (MCF-7) were washed with DPBS solution (Biowest) and then detached from the culture bottle with trypsin (Biowest). The cells were centrifuged and then resuspended in 1 ml of medium. The cell suspensions density was established based on calculations in Thoma chambers and diluted to obtain the appropriate cell density. Cell suspensions with a density of 10^6 cells/ml (in the case of fibroblasts) or 10^6 cells/ml, 2 x 10^6 cells/ml and 3 × 10^6 cells/ml (in the case of cancer cells) were prepared. Then, non-malignant cells were introduced into the microsystem, and after 24 h (when the fibroblasts had adhered), the cancer cell suspensions were introduced into the microsystem. The three different ratios of the seeding density of fibroblasts and cancer cells in the microsystem were compared: 1:1, 1:2 and 1:3. The three cell seeding density ratios for testing were selected from the literature data [1–3]. The cell co-cultures obtained in the microsystem were monitored using an inverted optical microscope (Olympus IX71) 24 h after seeding the cancer cells.

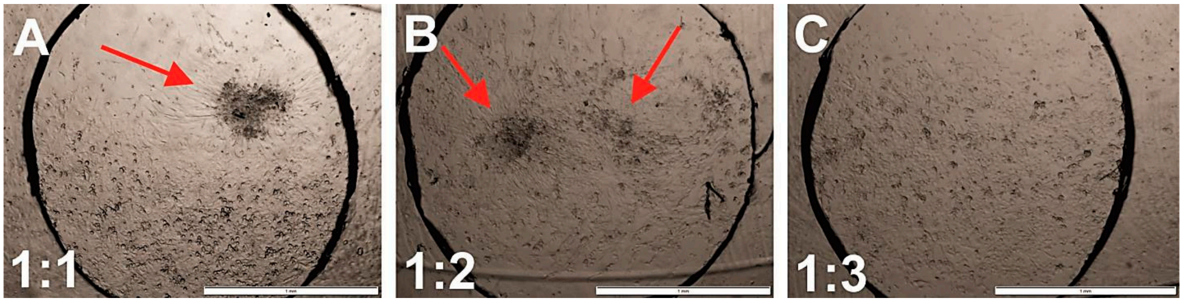

Figure S1. The co-cultures of fibroblasts and breast cancer cells in the microwell of the microsystem. Cell suspensions of different densities were introduced into the microsystem. The figure shows the co-cultures of HMF and MCF-7 cells in a 1:1 (A), 1:2 (B) and 1:3 (C) ratios.

In the case of co-culture of non-malignant and cancer cells in the ratio of 1:1 and 1:2, the formation of cell aggregates was observed due to the affinity of fibroblasts to cancer cells. The migration of cell aggregates in culture was also observed (Figure S1 A, B). The cell distribution was irregular. In the culture, clusters of cells were formed, which could be places with limited availability of oxygen and nutrients. In the case of co-culture of fibroblasts and cancer cells in the ratio of 1:3: no cell aggregates were formed in the culture and the distribution of cells in the culture microwell was regular (Figure S1 C). The advantage of the number of cancer cells over the number of non-malignant cells is similar to the physiological conditions, therefore this ratio of the seeding density was selected for further studies. It has been proved that the ratio of stromal cells in *in vivo* cancer tissue in patients ranges from 20 % to 90 % [2]. In the literature, in the case of cell co-cultures, different ratios of the density of the two types of cultured cells are tested, most often in the range between 1: 1 and 1:10 [3-6].

Comparison of the cell seeding in three culture microwells in a row of the microsystem

In order to check how the cells introduced into the microsystem are distributed in all microwells of the developed microsystem and to confirm the formation of a cell multilayer in each of the six culture microchambers, the additional staining of cells in the microsystem was performed. The non-malignant cells (HMF) were stained with the red CMTPX dye. MCF-7 cells were stained with CMFDA, which showed green fluorescence. 24 h after the introduction of the cancer cells into the microsystem a Z-axis scan of cell culture was performed using a confocal microscope (Zeiss Axio Observer 7 with LSM 900). Image acquisition was performed using ZEN Blue software. The cell imaging results are shown in Figure S2.

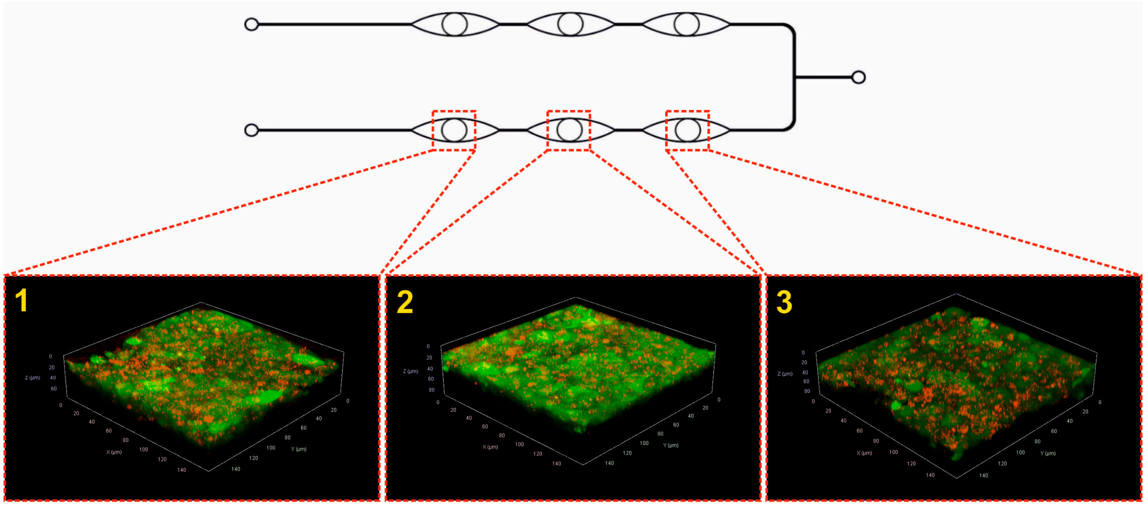

Figure S2. Imaging of cell multilayers obtained in three following microwells in a row of the microsystem (1,2,3 - numbers of following culture microwells in a row).

The cells were introduced into the microsystem at a flow rate of 10 µl/min. Thanks to the high introducing flow rate, the cells were regularly introduced over all the culture microwells and they fell to their bottom. According to the initial assumption, confirmed by computer flow simulations [7], the cells did not stop only in the initial culture microwells, but also reached the last microwells in the row, creating repeatable 3D cell microstructures (Figure S2).

2. Analysis of doxorubicin cytotoxicity on breast cells

In the first stage of the analysis of the effectiveness of combined therapy on a 3D model in the microsystem, the cytotoxicity of cytostatic agent (doxorubicin, DOX) on non-malignant and cancer breast cells was checked. The DOX cytotoxicity studies aimed to select the concentration of the cytostatic for combination therapy, with a selective effect on cancer cells.

Fibroblasts and cancer cell suspensions were plated in 96-well plates at a density of 10^4 cell/ml. Then, 1 mM stock of doxorubicin (DOX) was prepared and diluted in DMEM culture medium without phenol red. In this way, working solutions in concentrations: 0 μM, 0.5 μM, 1 μM, 2 μM, 3 μM, 4 μM and 5 μM were obtained. The obtained DOX solutions were introduced to the cells, previously washed with PBS, in a 96-well plate (100 μl of solution per well). The cells were incubated with DOX solutions for 24 h (37°C, 5 % CO_2), and then the cytotoxicity test (MTT assay according to the manufacturer procedure) was performed on non-malignant and cancer cells.

The obtained results are presented in Figure S3. It was observed that the viability of HMF and MCF-7 cells after incubation with a 1 μM solution of doxorubicin was high (approximately 100 % relative to the control) (Figure S3). It means that 1 μM DOX is the lowest (among the tested) non-toxic concentration of doxorubicin, and it was chosen for sequential photochemotherapy. A higher DOX concentration (3 μM) was also selected for combination therapy. DOX at this concentration was selectively cytotoxic to breast cancer cells and caused a slight decrease in cell viability (Figure S3). It was observed that cell viability did not always decrease with increasing test drug concentration, *eg*. the viability of both tested cell lines was higher at 1 μM DOX than 0.5 μM DOX. The reasons for this may be related to the mechanism of action of the drug, which interacts with the cell's DNA and inhibits its synthesis, *e.g.* it can be connected with unspecific inhibition of regulatory or feedback loops in signal pathways. The control sample consisted of HMF and MCF-7 cells incubated for 24 h with the culture medium without compound (0 μM DOX).

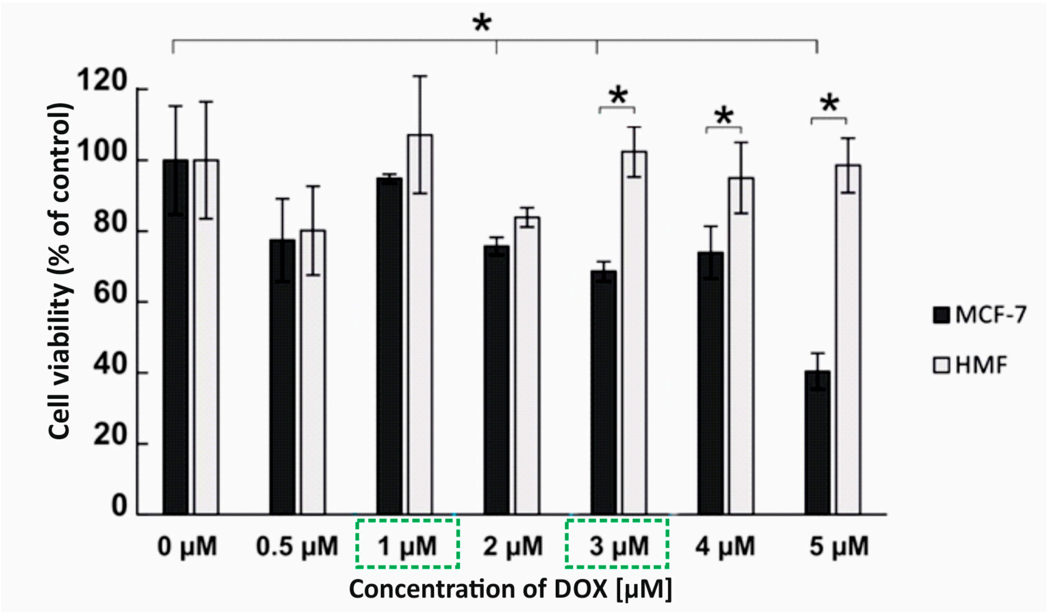

Figure S3. A viability of fibroblasts and breast cancer cells after incubation with doxorubicin solutions. Study performed in the macroscale. Doxorubicin in concentrations of 1 μM and 3 μM has been selected to perform combined photochemotherapy. Asterisks indicate statistically significant differences (ANOVA, α = 0.05).

In the next stage of the research, it was checked whether the cytotoxic effect caused by the action of doxorubicin, intercalating with DNA and inhibiting replication processes, would be enhanced by photodynamic therapy based on the activation of the photosensitizer (*meso*-tetraphenylporphyrin) accumulated in the cells.

References

[1] Y. Yang, X. Yang, J. Zou, C. Jia, Y. Hu, H. Du, H. Wang, Evaluation of photodynamic therapy efficiency using an in vitro three-dimensional microfluidic breast cancer tissue model. Lab Chip. 2015, 15, 735-744.

[2] W.E. Mesker, J.M.C. Junggeburt, K. Szuhai, P. de Heer, H. Morreau, H. J. Tanke, R.A.E.M. Tollenaar, The carcinoma-stromal ratio of colon carcinoma is an independent factor for survival compared to lymph node status and tumor stage. Cell Oncol., 2007, 29, 387–398.

[3] S.Y. Jeong, J.H. Lee, Y. Shin, S. Chung, H.J. Kuh, Co-Culture of Tumor Spheroids and Fibroblasts in a Collagen Matrix Incorporated Microfluidic Chip Mimics Reciprocal Activation in Solid Tumor Microenvironment. PLoS One. 2016, 11, e0159013.

[4] S-A. Kim, E.K. Lee, H-J. Kuh, Co-culture of 3D tumor spheroids with fibroblasts as a model for epithelialmesenchymal transition in vitro. Exp Cell Res. 2015, 335, 187–196.

[5] K. Yao, W. Li, K. Li, Q. Wu, Y. Gu, L. Zhao, Y. Zhang, X. Gao, Simple Fabrication of Multicomponent Heterogeneous Fibers for Cell Co-Culture via Microfluidic Spinning, Macromol Biosci. 2020, 20, e1900395.

[6] M. Nikkhah, J.S. Strobl, E.M. Schmelz, P.C. Roberts, H. Zhou, M. Agah, MCF10A and MDA-MB-231 human breast basal epithelial cell co-culture in silicon micro-arrays. Biomaterials, 2011, 32, 7625-7632.

[7] M. Flont, E. Jastrzębska, Z. Brzózka, Synergistic effect of the combination therapy on ovarian cancer cells under microfluidic conditions. Anal Chim Acta, 2020, 1100, 138., Analytica Chimica Acta, 1100, 2020, 138-148.
